# Comprehensive analysis of ferroptosis-related genes reveals potential therapeutic targets in osteoporosis patients: a computational analysis and *in vitro* experiments

**DOI:** 10.3389/fgene.2024.1522809

**Published:** 2025-01-10

**Authors:** Sihui Chen, Yi Jiang, Guoqin Xie, Peng Wu, Jinyu Zhu

**Affiliations:** ^1^ Department of Orthopedics, First Hospital of Jiaxing, Jiaxing, China; ^2^ College of Medicine, Jiaxing University, Jiaxing, China

**Keywords:** KMT2D, osteoporosis, ferroptosis, aging, BMSCs

## Abstract

**Background:**

Ferroptosis-related genes have been reported to play important roles in many diseases, but their molecular mechanisms in osteoporosis have not been elucidated.

**Methods:**

Based on two independent GEO datasets (GSE35956 and GSE35958), and GSE35959 as the validation dataset, we comprehensively elucidated the pathological mechanism of ferroptosis-related genes in osteoporosis by GO analyses, KEGG analyses and a PPI network. Then, We used Western Blot (WB) and Quantitative real-time polymerase chain reaction (qPCR) to verify the expression level of KMT2D, a ferroptosis-related hub gene, in clinical samples. Subsequently, we predicted the upstream miRNA of KMT2D gene and analyzed the mechanism of KMT2D in osteoporosis, the potential prognostic value and its immune invasion of KMT2D in pan-cancer.

**Results:**

This study identified KMT2D and MYCN, TP63, RELA, SOX2, and CDKN1A as key ferroptosis-related genes in osteoporotic cell aging. The independent dataset validated that the expression level of KMT2D was significantly upregulated in osteoporosis samples. The experimental verification results of qPCR and WB indicate that KMT2D is highly expressed in patients with osteoporosis. Further analysis revealed that the hsa-miR-204-5p-KMT2D axis may play an important role in the aging of osteoporotic cells. The analysis of KMT2D reveals that KMT2D may mainly play a role in the aging of osteoporotic cells through epigenetics and the value in pan-cancer.

**Conclusion:**

The study provides a theoretical basis for the treatment of osteoporosis.

## 1 Introduction

The aging of the global population is contributing to an increase in osteoporosis, which is a serious public health issue (Lane, 2006). Osteoporosis and its associated complications are common causes of morbidity and mortality in older adults (Johnston and Dagar, 2020). Although many different factors contribute to bone mass loss as aging, age-related bone loss is generally accepted to be associated with an imbalance between osteoblast-mediated bone creation and osteoclast-mediated bone breakdown resorption (Al-Bari and Al, 2020). Osteoporosis treatment drugs are divided into three categories: bone resorption inhibitors (bisphosphonates, estrogens, selective estrogen receptor modulators, calcitonins, isoflavones), bone formation promoters (parathyroid hormone drugs, anabolic hormones, vitamin K2), and bone metabolism regulators (calcium, active vitamin D3). Mesenchymal stem cells (BMSCs) from patients with osteoporosis have the capacity for self-renewal and act as a permanent reservoir for the production of somatic cells (Lin et al., 2019; Veronesi et al., 2011). Studies have shown that bone diseases such as aging and osteoporosis affect BMSCs’ self-renewal ability and differentiation potential (Pignolo et al., 2019; Chandra and Rajawat, 2021; Farr and Khosla, 2019). It has been discovered that BMSCs’ osteogenic differentiation declines with aging, which puts a cap on their ability to regenerate bone (Abdelmagid et al., 2015). The dynamic process of senescence in human Mesenchymal Stem Cells (MSCs) is accompanied by several signaling pathways, genetic and epigenetic regulation of the transcriptome, and functional changes in metabolism (Mani et al., 2020; Hu M. et al., 2022).

Ferroptosis has been reported to be involved in the development and progression of many diseases (Proneth and Conrad, 2019; Bayır et al., 2020). There are studies reporting ferroptosis is related to the occurrence and development of osteoporosis, and its regulation can effectively prevent osteoporosis (Liu et al., 2022; Gao et al., 2022). The onset of osteoporosis is often associated with hormonal imbalance, nutritional factors, genetic factors, especially aging. Notable features of ferroptosis are iron overload and increased ROS, both of which can affect the progression of osteoporosis. Ferroptosis-related genes have been reported to play important roles in many diseases, but their molecular mechanisms in osteoporosis have not been elucidated. Therefore, it is necessary to clarify the pathophysiology and mechanisms of ferroptosis-related genes and osteoporosis during aging.

As a result of the advent of high-throughput screening technologies, gene microarray analysis has developed into an effective tool for identifying differentially expressed genes (DEGs), which may propose possible biomarkers in various diseases (Peng et al., 2020; Hu Y. et al., 2022). In this study, we conducted a comprehensive evaluation of the roles of ferroptosis-related genes in the regulation and associations of cellular senescence and osteogenic differentiation based on GSE35956, GSE35958, and GSE35959 datasets with MSCs. Then, the KMT2D was identified as a key driver gene of ferroptosis for the aging process in BMSCs by verifying clinical samples. Finally, we performed miRNA-gene and protein interaction analyses on key Hub genes in aging osteoporosis. The study provides a theoretical basis for the treatment of osteoporosis. Meanwhile, osteoporosis has been reported to be closely associated with cancer (Zhang et al., 2023), therefore, we conducted further pan cancer analysis.

## 2 Materials and methods

### 2.1 Microarray data collection and processing

**FIGURE 1:**
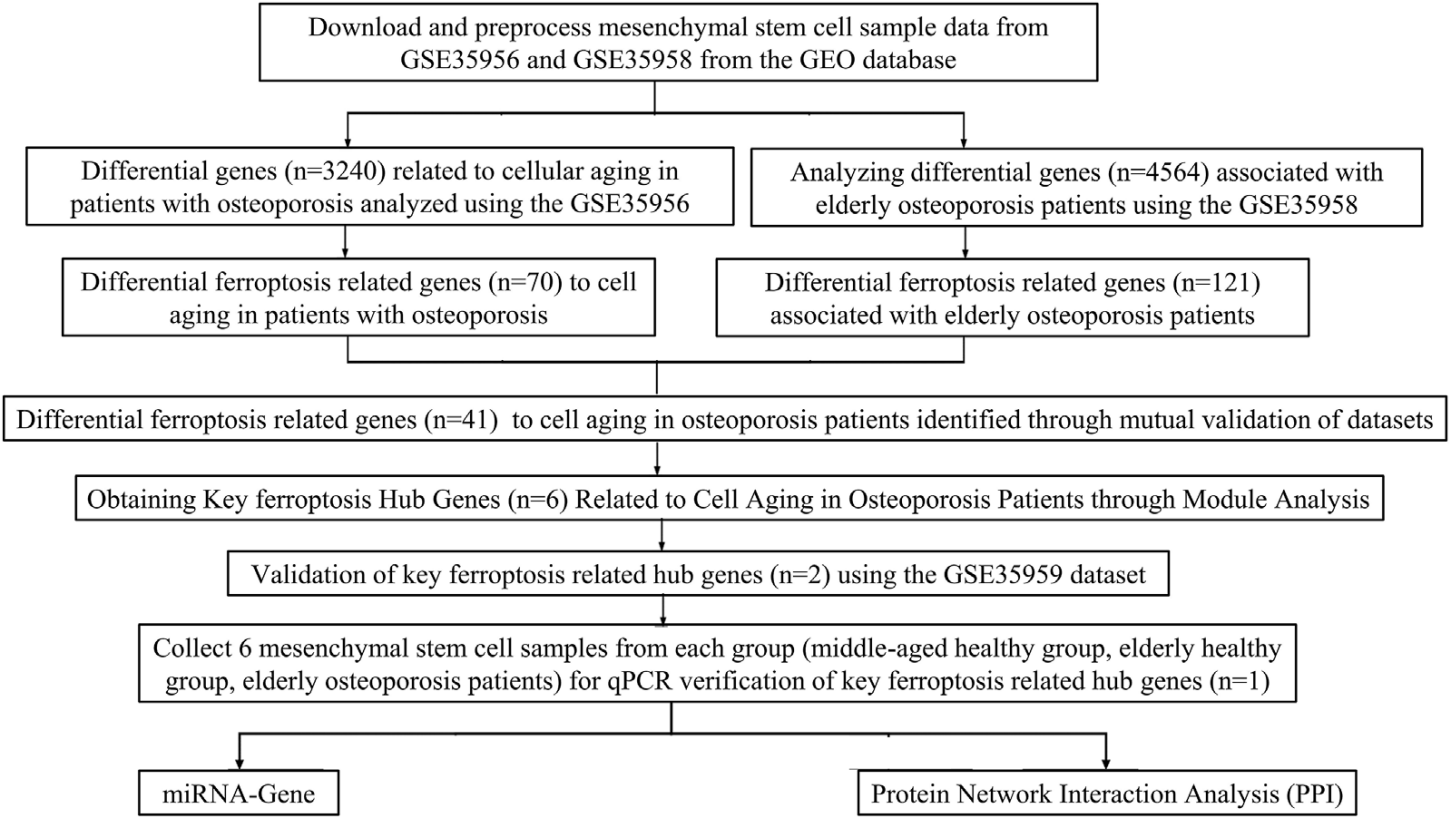
The flowchart of the present study design.

The research and design roadmap is shown in Figure 1. Gene expression microarray datasets, including GSE35958 and GSE35956, were downloaded from Gene Expression Omnibus (GEO) (https://www.ncbi.nlm.nih.gov/geo/). GSE35958 was obtained from the GPL570 platform. It included five elderly patients with osteoporosis (The average age of the elderly osteoporosis group was 86.2 years, with five females) who had isolated human mesenchymal stem cells from the femoral head after experiencing a low-energy fracture in the femoral neck, four elderly patients with non-osteoporosis (The average age of the elderly non-osteoporotic group was 81.75 years, with three females and one male) who isolated mesenchymal stem cells from the femoral head after undergoing total hip arthroplasty. GSE35956 was obtained from the GPL570 platform, including five cases of bone marrow mesenchymal stem cells isolated from the femoral head in elderly osteoporotic patients (The mean age of the osteoporosis group was 86.2 years, with five females) after low-energy femoral neck fractures, and five cases of bone marrow mesenchymal stem cells from middle-aged non-osteoporotic donors after total hip arthroplasty (The average age of the middle-aged non-osteoporotic group was 57.6 years, with four females and one male). GSE35959 was obtained from the GPL570 platform, including four elderly non-osteoporotic (The average age was 81.75 years, with three females and one male), 10 middle-aged non-osteoporotic (The average age is 57 years, with seven females and three males), and five elderly osteoporotic patients (The average age was 86.2 years, with five females). The BMSCs were isolated from those patients’ femoral canals after low-energy femoral neck fractures. We used the R4.2.1 (R: A language and environment for statistical computing. R Foundation for Statistical Computing, Vienna, Austria. URL https://www.R-project.org/) tool to evaluate gene expression levels by analyzing raw data from microarrays (Su et al., 2021).

### 2.2 GEO database used to screen differentially expressed genes in osteoporosis patients

To evaluate the expression level of ferroptosis-related genes in regulating the aging process of osteoporosis, GEO data GSE35958 and GSE35956 were used for gene expression data analysis. Specifically, data standardization testing was performed first. If there is no batch effect in the data, it can be used as a batch of data for subsequent analysis. Use Fold change and adjusted p-values to select differentially expressed genes (FC > 1.5 or <0.67 and P < 0.05 are considered to have significant differences in genes) (Sun et al., 2019). From the FerrDb database (FerrDb: http://www.zhounan.org/ferrdb/current/) Screening for ferroptosis-related genes in PubMed, Google Scholar, and KEGG pathways (Sun et al., 2019). Next, we used a Venn map and enrichment analysis to explore the common molecular mechanism of ferroptosis-related genes in osteoporosis.

### 2.3 Functional and pathway enrichment analysis

Metascape (https://metascape.org/gp/index.html#/main/step1) is a website for analyzing gene or protein lists, used to analyze functional clustering of gene sets. The R package ClusterProfiler package is used to analyze the gene ontology (GO) and the gene set of the Kyoto Encyclopedia of Genes and Genomes (KEGG), and P < 0.05 is considered significant (Zhou et al., 2019).

### 2.4 Analysis of the Hub gene for key ferroptosis-related genes in osteoporosis patients

Utilizing the STRING database (https://string-db.org/) to obtain the network diagram of ferroptosis-related genes and obtain the Hub gene module through the MCODE plugin in Cytoscape software. Exploring key Hub genes related to ferroptosis through Venn diagrams. Subsequently, the GSE35959 dataset was used to validate key ferroptosis genes during the aging process of osteoporotic cells (Doncheva et al., 2019).

### 2.5 Clinical sample collection

This study was approved by the Medical Ethics Committee of the First Hospital of Jiaxing (2023-LY-069). We made sure to obtain informed consent from all participants or their legal guardians. Recruit patients with femoral fractures or requiring hip-knee replacement, record the general condition of the patient, preoperative and postoperative vital signs, clinical laboratory tests, and imaging tests. Our inclusion criteria for patients with osteoporosis were based on DXA (dual-energy X-ray absorptiometry). According to T-score and Z-score, patients were divided into the elderly osteoporosis group (T-score ≦ −2.5, Age > 60), elderly normal group (T-score ≧ −1.0, Age > 60), and middle-aged normal group (Z-score > −2.0, Age 30–60). A total of three groups, each group of six people. Residual bone marrow tissue on the reamer was collected during surgery, about 1∼2 mL. The exclusion criteria and inclusion criteria for our research are presented in Table 1.

**TABLE 1:** Exclusion criteria and inclusion criteria in the present study.

Exclusion criteria	
	Severe heart, lung, kidney, liver insufficiency, and other diseases, cannot be tolerated
	Underlying medical conditions such as severe hypertension, diabetes, immunocompromise, nodules, tumors, etc
	History of hormonal use ≧ 3 months
	Patients with secondary osteoporosis
Inclusion criteria	
	Patients with surgical indications of femoral fractures or requiring hip-knee replacement
	Patients have no contraindications to surgery, such as local infection and coagulation abnormalities
	Patients were not treated with anti-osteoporosis

### 2.6 Isolation and culture of bone mesenchymal stem cells

Tissue samples comprising 1∼2 mL were washed three times with sterile phosphate-buffered saline (PBS; Solarbio, Beijing, China), then were suspended in DMEM/F12 (Procell, PM150312, China) containing 10% fetal bovine serum (FBS; Thermo Fisher Scientific Inc, 26010074, United States), 1% penicillin and 1% streptomycin (Thermo Fisher Scientific Inc, 15140148, United States), incubated at 37°C in a humidified atmosphere containing 5% CO_2_. Every three days, the medium was changed, and when the tissue block cells reached 80% confluence, they were passaged. An inverted optical micro-scope (CKX41, Olympus Corporation, Japan) was used to observe the morphology of MSCs. For additional research, MSCs were resuspended in DMEM/F12 at the desired density and put through several experiments.

### 2.7 Flow cytometry to identify bone mesenchymal stem cells

When the P3 BMSCs were cultured to close to confluency, the medium and washed twice with PBS, digested 0.25% trypsin for 2 min, rinsed twice with PBS, added 1 mL PBS to create 5 × 106/mL single-cell suspension, five samples were made. Cell suspensions are dropwise with fluorescently labeled mice anti-human monoclonal antibodies CD29-FITC (Solarbio, K010108M, China), CD90-PE (Solarbio, K010110M, China), CD34-FITC (Solarbio, K010109M, China), CD45-PE/CY7 (Solarbio, K010111M, China) and blank control with nothing added. Incubated at 4°C protected from light for 30 min and washed PBS twice. After adding the FITC-labeled mouse anti-human IgG monoclonal antibodies (Solarbio, K21001M-FITC, China), PE-labeled murine anti-human IgG monoclonal antibody (Solarbio, K2001M-PE, China), PE-Cy7-labeled murine anti-human IgG monoclonal antibody (Solarbio, K2001M-PE-Cy7, China) incubated at 4°C in the dark for 30 min, washed PBS twice, resuspend cells with 0.5 mL PBS, and detected by flow cytometry.

### 2.8 Determination of ferroptosis-related Hub genes expression by qPCR

Quantitative real-time PCR (qPCR) was used to detect the changes in the expression level of ferroptosis-related Hub genes (GAPDH as an internal reference). To ensure the reproducibility of the results, we set six replicates for each group. The specific operation is as follows: the bone marrow-derived mesenchymal stem cells (BMSCs) were washed twice by sterile PBS. Then 1 mL Trizol (Thermo Fisher Scientific Inc, 15596026, United States) was added to the flask to cleave BMSCs. Then the cell lysate was removed from the flask with a pipette and ice bath for 5 min, 0.2 mL chloroform was added and vigorously inverted for 15 s. Ice bath for 10 min and centrifuged at 12,000 rpm at 4°C for 15 min. Pipetted the upper layer into a new EP tube, mixed with 0.5 mL of isopropanol, left at room temperature for 10 min, and centrifuged at 12,000 rpm at 4°C for 10 min. After removing the supernatant, wash the RNA with 75% ethanol and mix the sample by shaking. Centrifuged at 12,000 rpm for 5 min, the supernatant was aspirated and the ethanol was evaporated. Purified RNA was eluted by DNase/RNase-Free Water (Beyotime, Shanghai, China), and the integrity of the RNA was confirmed by 1% agarose gel electrophoresis. RNA quantity and purity were determined by a Microvolume UV-Vis Spectrophotometer (NanoDrop One, Thermo Fisher Scientific, United States).

### 2.9 Western blot

Western blot analysis was used to measure KMT2D expression. Firstly, total cell lysates were separated by sodium dodecyl sulfate (SDS)-polyacrylamide gel electrophoresis (PAGE) and transferred to polyvinylidene fluoride (PVDF) membranes. Next, membranes were blocked at room temperature with 5% defatted milk for 1 h. After incubating the membrane with anti-KMT2D (Abcam, ab213721, China) and anti-GAPDH (Biosharp, BL006B, China) overnight at 4°C, the process was completed. After being washed three times with tris-buffer saline and Tween (TBST) for 10 min each, the membranes were incubated with corresponding horseradish peroxidase-conjugated second antibodies. Immunoreactivity was detected by enhancing the chemiluminescence reaction.

### 2.10 Prediction of upstream miRNA in KMT2D

To evaluate the upstream mechanism of key Hub genes in osteoporotic mesenchymal stem cells, the TargetScanHuman database (https://www.targetscan.org/vert_72/) was first used to predict miRNA binding sites. The TargetScanHuman database is a software that has excellent performance in predicting miRNA binding sites in mammals. The lower the context++score, the greater the probability that the site is a target. In addition, the percentage is the conversion of a score, and the closer the value is to 100, the greater the probability that the site is a true target. Based on the Human microRNA Disease Database (HMDD), which provides miRNAs with abnormal regulation in various human diseases, miRNAs related to osteoporosis were found. The HMDD database is a comprehensive resource database, and the data collected in the database are all disease-related miRNAs supported by experiments. Finally, it has been validated in starBase v2.0 (https://rnasysu.com/encori) and has been validated by existing literature (Tang et al., 2022).

### 2.11 Mechanism analysis at the protein level of KMT2D in osteoporosis

To investigate the protein interaction mechanism of key Hub genes in the aging process of osteoporotic mesenchymal stem cells, we used GeneMANIA (https://genemania.org/). A PPI network centered around key Hub genes was constructed, which includes association data on protein genetic interactions, pathways, co-expression, co-localization, and protein domain similarity. Then, GO functional enrichment and KEGG pathway analysis were performed on genes constructed using GeneMANIA centered around the Hub gene (Ding et al., 2021).

### 2.12 Differential expression and potential prognostic value of KMT2D in pan-cancer

Based on TCGA database, we analyzed KMT2D's pan-cancer expression level. Gene Expression Profiling Interaction Analysis (GEPIA, http://gepia.cancer-pku.cn/index.html) can be used to evaluate the KMT2D expression level of 9736 tumor samples and 8587 normal samples in the GTEx database and TCGA database. When obtaining KMT2D’s gene expression profile, Analysis of Variance method was used to compare with the following thresholds: q value cutof = 0.05 and | log2FC | cutof = 0.59 (Luo et al., 2023).

### 2.13 KMT2D and immune infiltration in pan-cancer

We investigated the correlation between KMT2D expression and immune cell infiltration levels, using the XCELL method to demonstrate the landscape of various immune cell infiltration associated with KMT2D, spearman correlation analysis was conducted between KMT2D and immun infiltration scores in pan-cancer tissues. The vertical axis represents different immune infiltration scores, horizontal axis represents different tumor tissues, different colors represent correlation coefficients, positive values represent positive correlation, negative values represent negative correlation, and the stronger the correlation, the darker the color. *p < 0.05, **p < 0.01, ***p < 0.001 and asterisks represent importance (*p) (Zhao et al., 2023).

### 2.14 Statistical analysis

As mentioned above, most statistical analyses of differential gene expression were conducted using R 4.2.1 (R: A language and environment for statistical computing. R Foundation for Statistical Computing, Vienna, Austria. URL https://www.R-project.org/) and online databases. For GSE35956, GSE35958 and GSE35959, student t-tests or rank sum tests are used for comparison between two groups, and FC values are obtained by the ratio of the average expression levels between the two groups (FC > 1.5 or FC < 0.67 and P < 0.05 are considered significant differences). TCGA data were screened for differentially expressed genes using the ANOVA method (q value cutof = 0.05 and | log2FC | cutof = 0.59 were considered significantly different). All relevant analyses were conducted using the Spearman method, and statistical plotting was performed using the ggplot2 and pheatmap packages of R software.

## 3 Results

### 3.1 GEO database used to screen differentially expressed genes in osteoporosis patients

**FIGURE 2:**
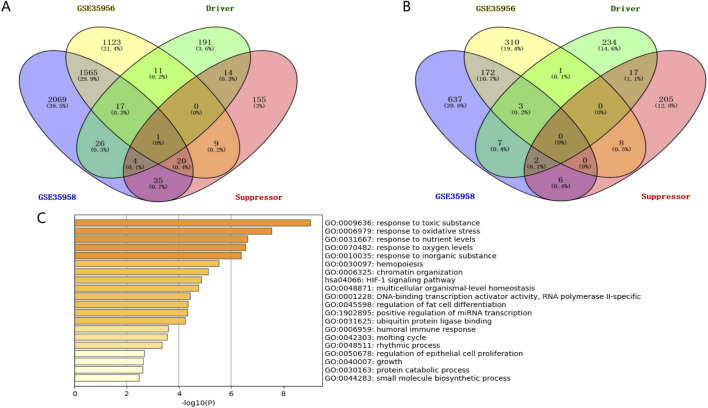
Identifying BMSCs from osteoporosis patients and non-osteoporotic donors (GSE35956 and GSE35958). **(A)** The screening results of significantly upregulated ferroptosis-related genes. **(B)** The screening results of significantly downregulated ferroptosis-related genes. **(C)** GO pathway and KEGG pathway enrichment results.

This study’s workflow is shown in Figure 1. Based on two datasets of bone mesenchymal stem cells (BMSCs) from osteoporotic and non-osteoporotic donors (GSE35956 and GSE35958). (the amount of genes)We found that 38 ferroptosis-related genes underwent significant upregulation (Figure 2A). Three ferroptosis-related genes underwent significant downregulation (Figure 2B). In addition, 41 differentially expressed ferroptosis-related genes are mainly enriched in the GO pathway and KEGG pathway (Figure 2C). The GO pathway mainly includes response to toxic substances, response to oxidative stress, response to nutrient levels, and response to oxygen levels, the KEGG pathway mainly includes the HIF-1 signaling pathway.

### 3.2 Analysis of the Hub gene for key ferroptosis-related genes in osteoporosis patients

**FIGURE 3:**
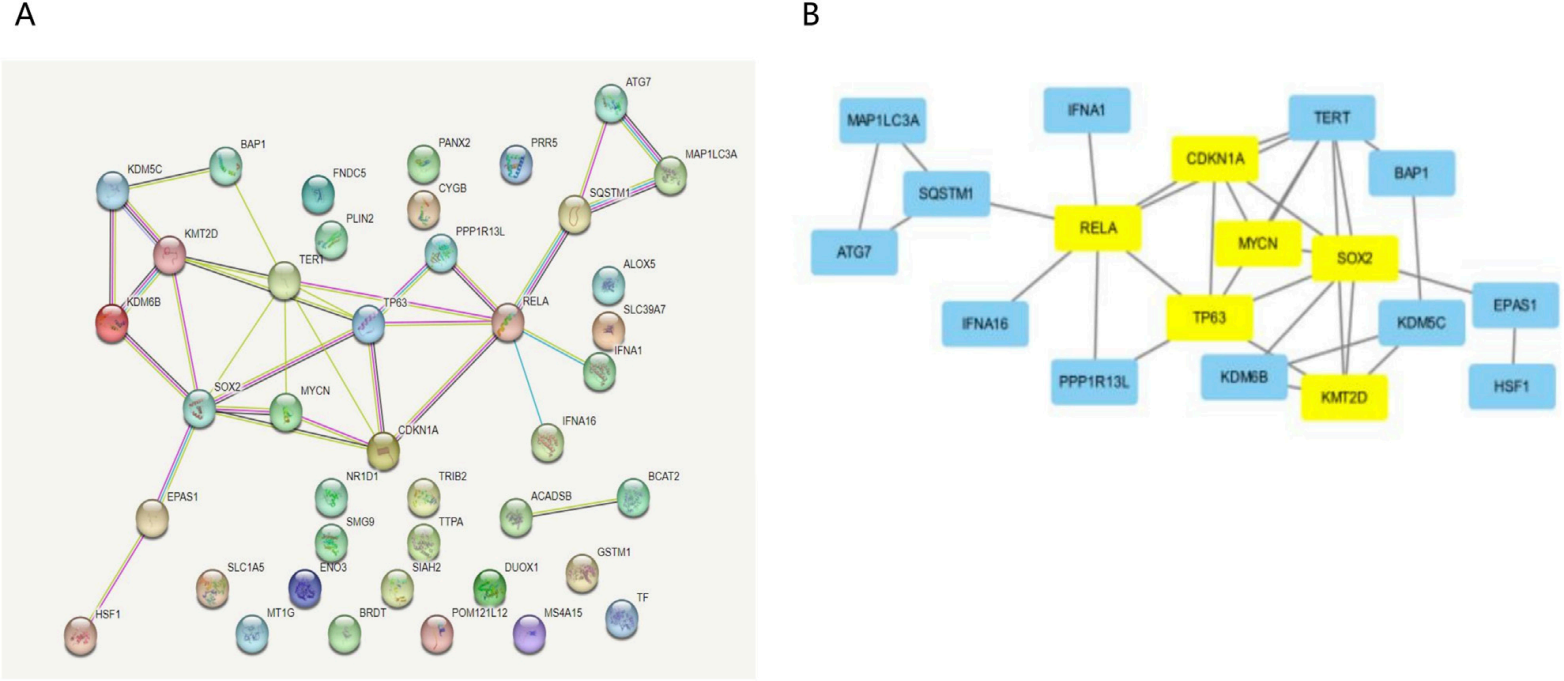
Analysis of the Hub gene for key ferroptosis during osteoporotic aging. **(A)** Build a PPI network diagram in a STRING database. **(B)** PPI network visualization by Cytoscape.

We constructed the PPI network in the STRING database, visualized it by Cytoscape (Figures 3A, B), and filtered critical subnets with the MCODE plugin. KMT2D, TP63, RELA, MYCN, SOX2, and CDKN1A were identified as the highest-scoring hub genes associated with ferroptosis-related genes during osteoporotic aging. KMT2D and MYCN were the driver genes of ferroptosis, TP63, RELA, SOX2, and CDKN1A were the inhibitory genes for ferroptosis.

### 3.3 GEO independent sample set validation of the expression levels of key ferroptosis-related genes in osteoporosis patients

**FIGURE 4:**
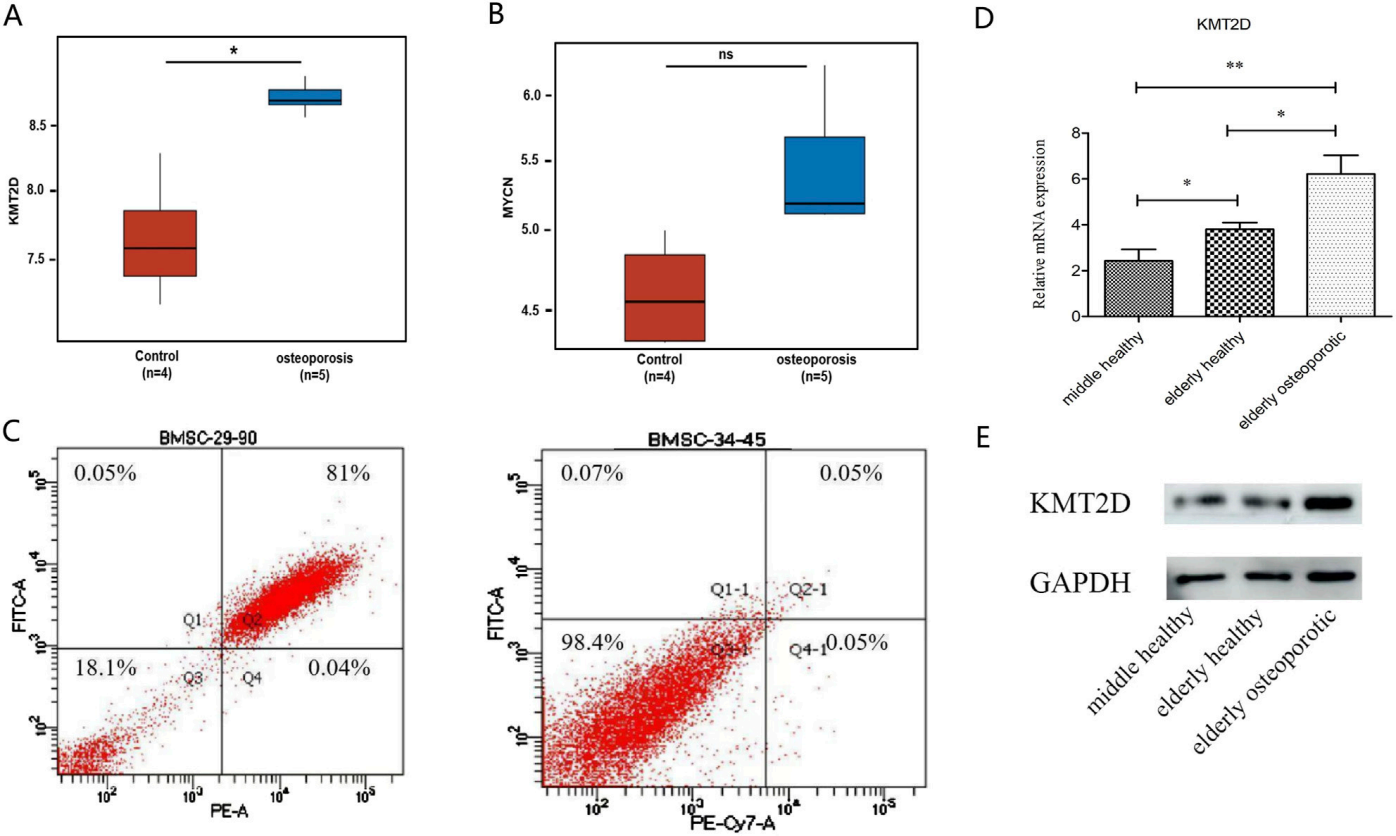
Validation of the key ferroptosis Hub gene. **(A)** Differential analysis of *KMT2D* expression between G1 (healthy) and G2 (osteoporotic) groups. **(B)** Differential analysis of *MYCN* expression between healthy and osteoporotic groups. **(C)** Identification of flow cytometry cells from cultured mesenchymal stem cells from primary isolation of clinical samples. **(D)** QPCR results validated by clinical samples. There was a significant difference among the three groups (P < 0.05). **(E)** Western blot results were confirmed by clinical samples, showing a significant difference between normal and osteoporotic groups.

To identify potential targets for treating osteoporosis, we have verified the ferroptosis-related KMT2D gene and MYCN gene, both of which are drivers of ferroptosis. The GSE35959 dataset has been analyzed. There was a significant difference in expression between KMT2D in the osteoporotic group compared to the healthy aging group (Figure 4A), but no significant difference in MYCN (Figure 4B).

### 3.4 Clinical independent sample set validation of the expression level of the key ferroptosis-related KMT2D gene in osteoporosis patients

The samples were as follows: six females in the elderly osteoporosis group, four females and two males in the elderly normal group, two females and four males in the middle-aged normal group. For clinical sample validation, Flow cytometry results showed that primary mesenchymal stem cells were successfully isolated from the sample (Figure 4C). The qPCR results showed that KMT2D had a significant difference among the three groups (P < 0.05) (Figure 4D). The Western blot results showed a significant difference between normal groups and osteoporotic groups (Figure 4E). All results suggest that KMT2D may be involved in the regulatory process of cellular senescence and osteoporosis. High expression of ferroptosis -related KMT2D gene may be a key gene regulating the aging process of osteoporotic cells.

### 3.5 Prediction of upstream miRNA in KMT2D gene

**FIGURE 5:**
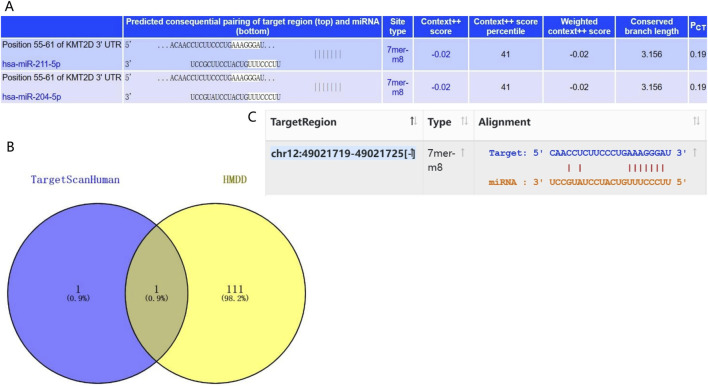
miRNA-gene interaction analysis. **(A)** The Target Scan Human database was used to predict the miRNAs with the most confidence in binding to *KMT2D*. **(B)** Based on the HMDD database, the Venn diagram represented that hsa-miR-204-5p was associated with osteoporosis and had the highest confidence in the combination with *KMT2D*. **(C)** Sequence binding of hsa-miR-204-5p and *KMT2D* was verified by the Starbase database.

To understand the upstream mechanism of KMT2D in osteoporotic aging, we utilized the Target Scan Human database to predict its miRNAs. Our analysis revealed that hsa-miR-211-5p and hsa-miR-204-5p have the highest probability of binding to KMT2D (Figure 5A). To further screen miRNAs that are regulated by key genes, 112 miRNAs were screened for association with osteoporosis based on the HMDD database. The Venn plot found that hsa-miR-204-5p was associated with osteoporosis and had the highest confidence in binding to KMT2D (Figure 5B). After extracting the sequences of hsa-miR-204-5p and KMT2D from the Starbase database, it was discovered that hsa-miR-204-5p binds to KMT2D. (Figure 5C).

### 3.6 Mechanism analysis at the protein level of KMT2D in osteoporosis

**FIGURE 6:**
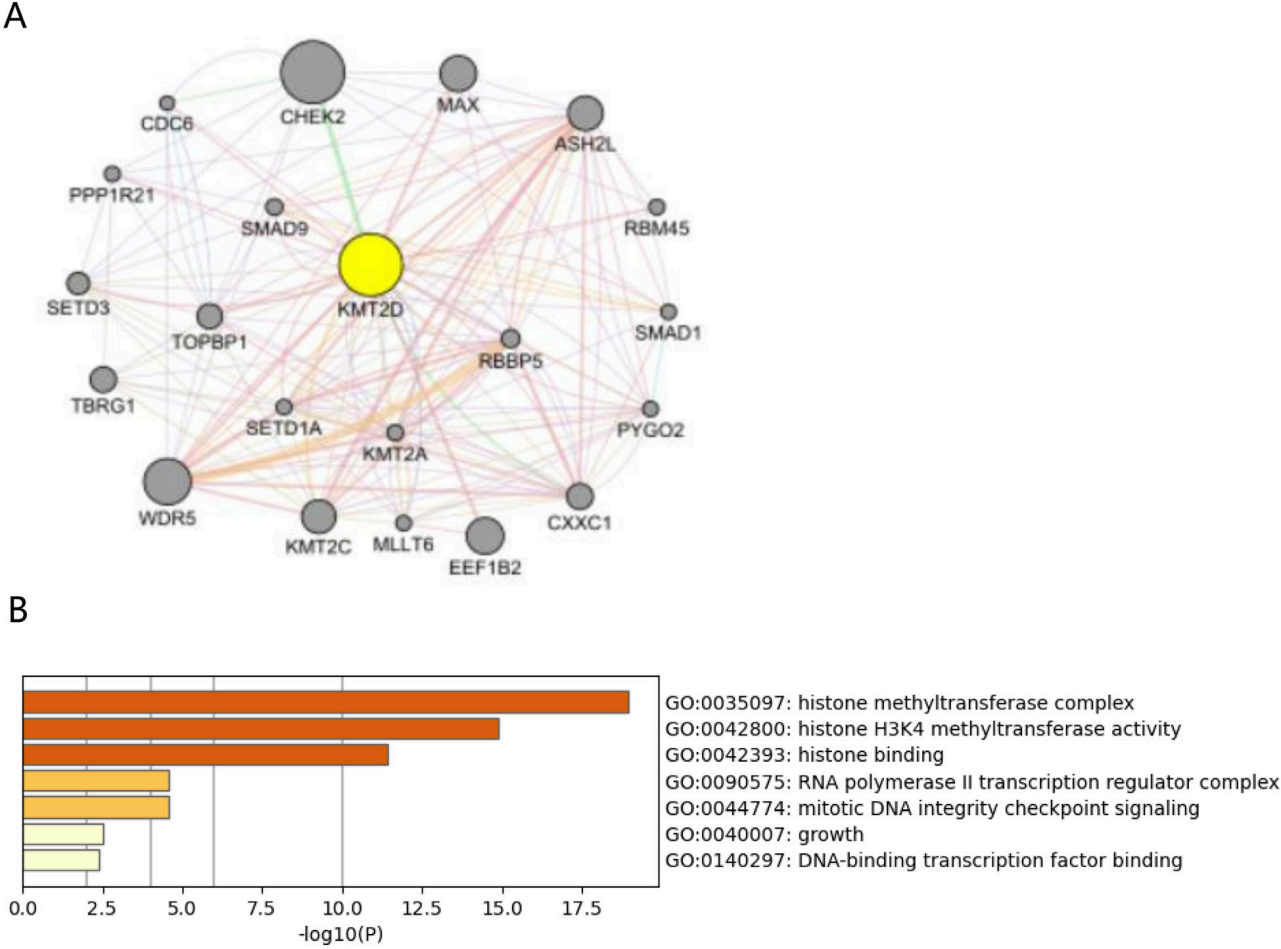
Protein interactions analysis of hub gene *KMT2D*. **(A)** PPI network plots of 21 genes centered on *KMT2D* were constructed using GeneMANIA. **(B)** GO functional enrichment was performed on these 21 genes, suggesting pathways in *KMT2D* may be involved.

A protein-protein interaction (PPI) network was created by GeneMANIA with 21 genes centered around KMT2D (Figure 6A). Functional enrichment analysis was conducted on these 21 genes, revealing significant enrichment in GO terminology related to Histone methyltransferase complex, Histone H3K4 methyltransferase activity, and Histone binding (Figure 6B). These findings suggest that KMT2D may primarily function through epigenetics.

### 3.7 Potential prognostic value of KMT2D in pan-cancer

**FIGURE 7:**
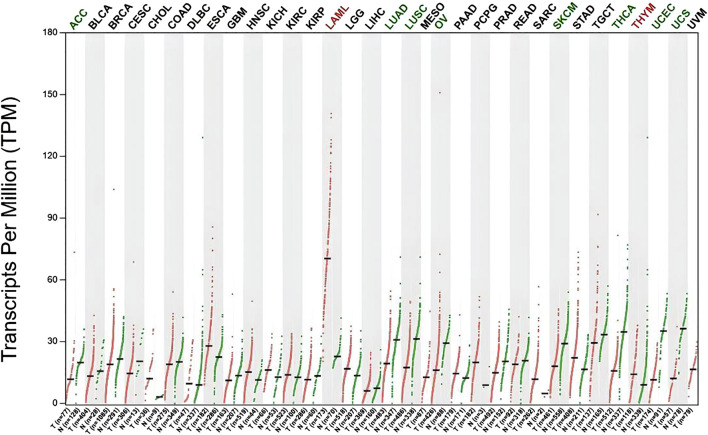
Differential expression of *KMT2D* in Pan-cancers. Differential expression of *KMT2D* in Pan-cancers (Green represents significant downregulation, while red represents significant upregulation). The types of cancer acronyms are as follows: ACC, adrenocortical carcinoma; LAML, acute myeloid leukemia; BLCA, bladder urothelial carcinoma; BRCA, breast invasive carcinoma; CESC, cervical squamous cell carcinoma; CHOL, cholangiocarcinoma; COAD, colon adenocarcinoma; DLBC, lymphoid neoplasm diffuse large B cell lymphoma; ESCA, esophageal carcinoma; GBM, glioblastoma multiforme; HNSC, head and neck squamous cell carcinoma; KICH, kidney chromophobe; KIRC, kidney renal clear cell carcinoma; KIRP, kidney renal papillary cell carcinoma; LGG, brain lower grade glioma; LIHC, liver hepatocellular carcinoma; LUAD, lung adenocarcinoma; LUSC, lung squamous cell carcinoma; MESO, mesothelioma; OV, ovarian serous cystadenocarcinoma; PAAD, pancreatic adenocarcinoma; PCPG, pheochromocytoma and paraganglioma; PRAD, prostate adenocarcinoma; READ, rectum adenocarcinoma; SARC, sarcoma; SKCM, skin cutaneous melanoma; STAD, stomach adenocarcinoma; TGCT, testicular germ cell tumors; THCA, thyroid carcinoma; THYM, thymoma; UCEC, uterine corpus endometrial carcinoma; UCS, uterine carcinosarcoma; UVM, uveal melanoma.

According to this result, KMT2D’s expression in a lot of cancers has changed significantly, KMT2D is significantly upregulated in Acute myeloid leukemia (AML) (Figure 7).

### 3.8 KMT2D and immune infiltration in pan-cancer

**FIGURE 8:**
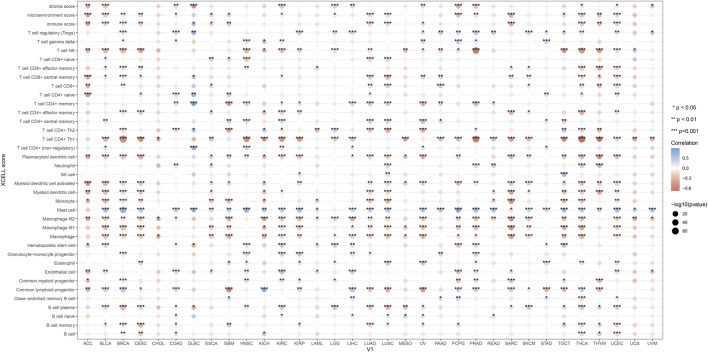
*KMT2D* and immune infiltration in pan-cancer. In order to investigate the immunological role of *KMT2D* in cancer environments, the estimated values of *KMT2D* in pan-cancer were calculated.

In order to investigate the immunological role of KMT2D in cancer environments, the estimated values of KMT2D in pan-cancer were calculated (Figure 8). As shown in Figure 8, We found that KMT2D was negatively correlated with immune invasion in most tumors, suggesting that KMT2D plays an important role in the cancer immune microenvironment, and the high expression of KMT2D inhibits the infiltration of immune cells, thereby reducing the anti-tumor immunity of cancer patients.

## 4 Discussion

Osteoporosis is a significant issue in the global public healthcare system, which affects around 200 million people worldwide. Osteoporosis and osteoporosis-related fractures are common causes of morbidity and mortality in older adults (Johnston and Dagar, 2020). Half of all postmenopausal women suffer from fractures related to osteoporosis, and 25% suffer from spinal deformities. Furthermore, 15% suffer from hip fractures at some point in their lifetime (Cotts and Cifu, 2018; Black and Rosen, 2016). Osteoporosis is a multifactorial disease, and the occurrence of osteoporosis is not only closely related to aging (Chandra and Rajawat, 2021; Khosla et al., 2022), but it is also related to atherosclerosis, intestinal microbiota, sarcopenia, and vascular mineralization (Khosla et al., 2022; Mo et al., 2022; Anagnostis et al., 2022; Laurent et al., 2019; Yang and Kim, 2021). The generation and development of osteoporosis are regulated by genetic factors and regulatory factors such as TGF-β, BMP, and FGF through multiple pathways, including the Wnt signaling pathway, the Notch signaling pathway, and the MAPK signaling pathway (Gao et al., 2023). These studies are expected to provide new perspectives and approaches for the diagnosis and treatment of osteoporosis.

We used GSE35956 and GSE35958 from GEO data, for gene expression data analysis. Following data correction, We found that 38 ferroptosis-related genes underwent significant upregulation and 3 ferroptosis-related genes underwent significant downregulation. GO and KEGG analyses revealed possible gene enrichment associated with osteoporosis and ferroptosis. By constructing the PPI network, KMT2D, TP63, RELA, MYCN, SOX2, and CDKN1A were selected as the hub genes. KMT2D and MYCN were the driver genes of ferroptosis, TP63, RELA, SOX2, and CDKN1A were the inhibitory genes for ferroptosis as they have been reported in many studies. The PI3K pathway plays a crucial role in regulating ER-dependent transcription in breast cancer. This pathway is controlled by an epigenetic regulator called KMT2D, which acts as a tumor suppressor gene (Toska et al., 2017). However, KMT2D can also interfere with the development of B cells and promote the development of lymphoma, lung cancer, and breast cancer (Zhang et al., 2015; Toska et al., 2017). There are currently a number of epigenetic modifiers used for common epigenetic modifications and treatments, and Vorinostat, Romidepsin, and Belinostat have been approved. Decitabine, Tazemetostat, and CPI-1205 are in clinical trials. They may play a role in epigenetic modification of KMT2D. However, there are no drugs that have clearly targeted KMT2D as a therapeutic target (Chebly et al., 2021). MYCN is responsible for TFRC-dependent ferroptosis and can lead to transcription-replication conflicts (Lu et al., 2021). The transcription factor and proto-oncogene MYCN is reviewed as a potential specific target for cancer therapy. Amplification of MYCN is frequently found in a number of advanced-stage tumours, including neuroblastoma (25%), small cell lung cancers (7%), alveolar rhabdomyosarcoma and retinoblastoma. MYCN reporter gene assay will be scaled up for high throughput screens of compound libraries and will aid in the future development of specific therapeutic strategies in neuroblastoma and other tumours (Papadopoulos et al., 2022; Lu et al., 2003). There are currently no drugs that explicitly MYCN as a therapeutic target. TP63 isoforms are involved in lineage specification, proliferation, differentiation, cell death, survival, DNA damage response, metabolism, and ferroptosis (Fisher et al., 2020; Wang et al., 2021). RELA regulates chondrocytes by inducing both anti-apoptotic and catabolic target genes in a biphasic manner (Kobayashi et al., 2016; Yu et al., 2020). Additionally, SOX2 is known to regulate various functions in cancer cells, including proliferation, apoptosis, invasion, migration, ferroptosis, and drug resistance (Ding et al., 2023; Novak et al., 2020). CDKN1A has also been identified as a key player in cell differentiation, migration, apoptosis, and ferroptosis (Bi et al., 2022; Kreis et al., 2019).

In our study, KMT2D had a high score in the PPI network and it was highly expressed in the bone marrow compared to other normal tissues as a driver gene of ferroptosis. We have identified KMT2D as a gene with potential for further validation. Our *in vitro* experiments have confirmed that KMT2D is a key driver of the aging process in osteoporotic cells, supporting our bioinformatics analysis.

We have concluded that high expression of ferroptosis -related KMT2D gene may be a key gene regulating the aging process of osteoporotic cells. However, the conclusion is experimentally validated only by clinical samples, which is a limitation of this study. It would be more convincing to provide more direct cell-based and animal evidence to demonstrate that KMT2D regulates cellular senescence through ferroptosis, thereby influencing osteoporotic diseases. This could be explored in future research.

Pan-cancer analysis found that KMT2D is significantly upregulated in AML, AML is a major blood stem cell disease. Research has shown that there is a close relationship between bone metabolism and hematopoietic stem cells within the bone hematopoietic niche. The relationship between AML and metabolic bone disease has received extensive research attention, which is an important factor seriously affecting the already impaired quality of life and survival of patients, posing a challenge to clinical treatment (Datzmann et al., 2018). Previous studies have suggested that patients with AML have an increased risk of developing osteoporosis, especially in elderly patients. It has been suggested that some susceptibility factors in AML may trigger the occurrence and development of osteoporosis (Brumariu et al., 2000). In previous studies, the genomic map of AML has been identified, which contains many biomarkers that may be related to the pathogenesis of osteoporosis (Arindrarto et al., 2021), suggesting that AML and osteoporosis share common genetic risk factors. However, so far, there have been no studies exploring the molecular mechanisms underlying the association between AML and osteoporosis based on bioinformatics analysis, and the co-pathogenesis of AML and osteoporosis is still unclear. Our study found that KMT2D is significantly upregulated in patients with osteoporosis and AML, indicating that KMT2D may be a potential therapeutic target for patients with osteoporosis and AML. However, further research and validation are needed.

## 5 Conclusion

Our study used multiple datasets to verify the differential expression of KMT2D in osteoporosis patients, and further verified the differential expression of KMT2D in osteoporosis patients through clinical samples, and elucidated the immune and pathological mechanisms of KMT2D in osteoporosis patients, and further analyzed the role of KMT2D in pan-cancer. In conclusion, based on bioinformatics analysis and *in vitro* experiments, we identified KMT2D as a promising ferroptosis driver gene during the aging process of BMSCs. Thus, it provides a theoretical basis for the treatment of senile osteoporosis.

## Data Availability

The data presented in the study are deposited in the Gene Expression Omnibus (GEO) (https://www.ncbi.nlm.nih.gov/geo/) repository, accession number GSE35956, GSE35958, and GSE35959.

## Abbreviations

BMSCs, Bone marrow mesenchymal stem cells; MSCs, Human mesenchymal stem cells; DEGs, Differentially expressed genes; GEO, Gene expression omnibus; GO, Gene ontology; KEGG, Kyoto encyclopedia of genes and genomes; DXA, Dual-energy X-ray absorptiometry; PBS, Phosphate-buffered saline; FBS, Fetal bovine serum; RNA, Ribonucleic acid; q-PCR, Quantitative real-time polymerase chain reaction; SDS, Sodium dodecyl sulfate; PAGE, Polyacrylamide gel electrophoresis; PVDF, Polyvinylidene fluoride; TBST, Tris-buffer saline and tween; HMDD, Human microRNA disease database; PPI, Protein-protein interaction.
