# Supplementary material for: Comprehensive analysis of ferroptosis-related genes reveals potential therapeutic targets in osteoporosis patients: a computational analysis and in vitro experiments
Source: Front Genet. 2025 Jan 10;15:1522809. doi: 10.3389/fgene.2024.1522809 (PMC11757248; doi:10.3389/fgene.2024.1522809)
Supplement: Supplementary file 1 [file Table1.docx]

**Table 1**. Exclusion criteria and inclusion criteria in the present study.

| Exclusion criteria |  |
| --- | --- |
|  | Severe heart, lung, kidney, liver insufficiency, and other diseases, cannot be tolerated |
|  | Underlying medical conditions such as severe hypertension, diabetes, immunocompromise, nodules, tumors, etc |
|  | History of hormonal use ≧ 3 months |
|  | Patients with secondary osteoporosis |
| Inclusion criteria |  |
|  | Patients with surgical indications of femoral fractures or requiring hip-knee replacement |
|  | Patients have no contraindications to surgery, such as local infection and coagulation abnormalities |
|  | Patients were not treated with anti-osteoporosis |
