# Supplementary material for: Comprehensive analysis of ferroptosis-related genes reveals potential therapeutic targets in osteoporosis patients: a computational analysis and in vitro experiments
Source: Front Genet. 2025 Jan 10;15:1522809. doi: 10.3389/fgene.2024.1522809 (PMC11757248; doi:10.3389/fgene.2024.1522809)
Supplement: Supplementary file 2 [file Table2.docx]

**Table 3.** Gene Sequence for Real-Time PCR Reaction.

| **gene** | **Nucleotide sequence (5’-3’)** |
| --- | --- |
| *RELA* | F: GTCATCCCTGAGCACCATCAACTAT |
|  | R: AGGCGAGTTATAGCCTCAGGGTACT |
| *CDKN1A* | F: CGTGAGCGATGGAACTTCGACTTTG |
|  | R: GAGGCACAAGGGTACAAGACAGTGA |
| *KMT2D* | F: AGATCAACAAGCAGACCAAGGTGGG |
|  | R: CTCTCAGGCACAGCCAAGTTATCCA |
| *GAPDH* | F: TCGACAGTCAGCCGCATCTTCTTTT |
|  | R: CATGGAATTTGCCATGGGTGGAATC |
