# Supplementary figures and images for: Comprehensive analysis of ferroptosis-related genes reveals potential therapeutic targets in osteoporosis patients: a computational analysis and in vitro experiments
Source: Front Genet. 2025 Jan 10;15:1522809. doi: 10.3389/fgene.2024.1522809 (PMC11757248; doi:10.3389/fgene.2024.1522809)

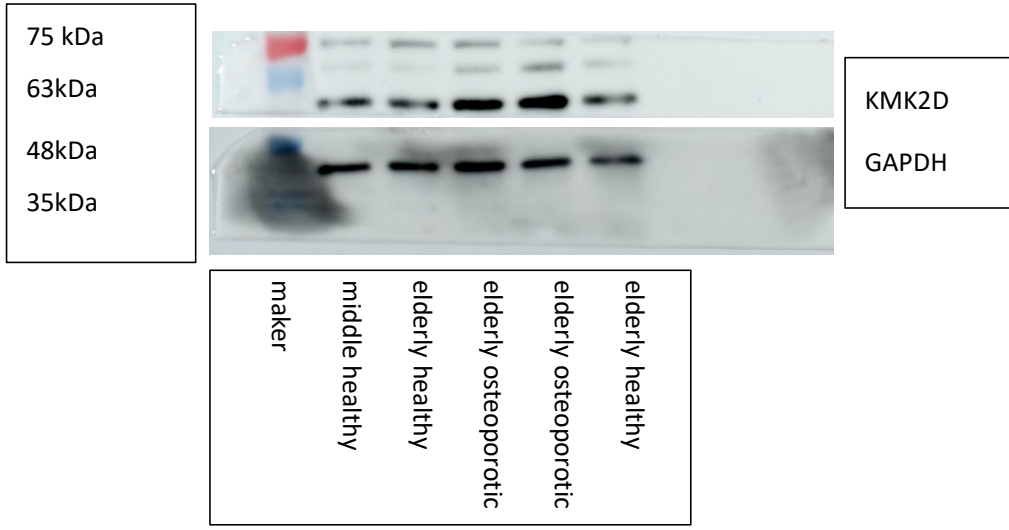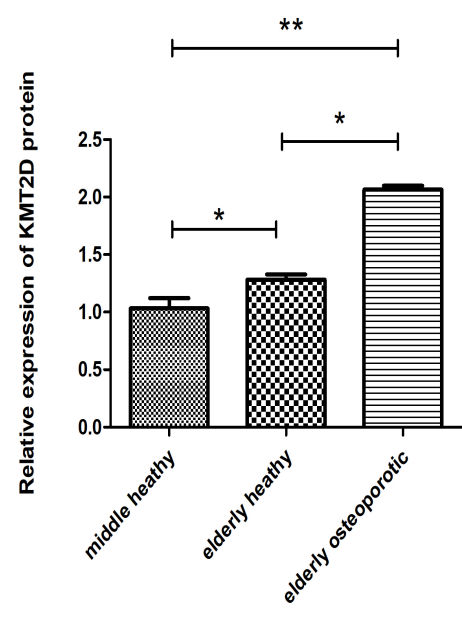

Supplement: Supplementary file 3 [file DataSheet1.pdf]
