# Supplementary material for: Comprehensive analysis of ferroptosis-related genes reveals potential therapeutic targets in osteoporosis patients: a computational analysis and in vitro experiments
Source: Front Genet. 2025 Jan 10;15:1522809. doi: 10.3389/fgene.2024.1522809 (PMC11757248; doi:10.3389/fgene.2024.1522809)
Supplement: Supplementary file 4 [file Table3.docx]

| GSE35956-C-donor1 （#276） | female | 42 yrs (middle-aged) |
| --- | --- | --- |
| GSE35956-C-donor2（#353） | female | 67 yrs (middle-aged) |
| GSE35956-C-donor3（#295） | male | 61 yrs (middle-aged) |
| GSE35956-C-donor4（#296） | female | 62 yrs (middle-aged) |
| GSE35956-C-donor5(#247) | female | 56 yrs (middle-aged) |
| GSE35956-osteopo_donor1(#535) | female | 79 yrs (elderly) |
| GSE35956-osteopo_donor2(#547) | female | 94 yrs (elderly) |
| GSE35956-osteopo_donor3(#558) | female | 87 yrs (elderly) |
| GSE35956-osteopo_donor4(#572) | female | 82 yrs (elderly) |
| GSE35956-osteopo_donor5(#573) | female | 89 yrs (elderly) |
| GSE35958--old_donor1(#520) | female | 79 yrs (elderly-aged) |
| GSE35958-old_donor2(#559) | male | 79 yrs (elderly-aged) |
| GSE35958-old_donor3(#606) | female | 80 yrs (elderly-aged) |
| GSE35958-old_donor4(#663) | female | 89 yrs (elderly-aged) |
| GSE35958-osteopo_donor1(#535) | female | 79 yrs (elderly) |
| GSE35958-osteopo_donor2(#547) | female | 94 yrs (elderly) |
| GSE35958-osteopo_donor3(#558) | female | 87 yrs (elderly) |
| GSE35958-osteopo_donor4(#572) | female | 82 yrs (elderly) |
| GSE35958-osteopo_donor5(#573) | female | 89 yrs (elderly) |
| GSE35959-C-donor1 （#276） | female | 42 yrs (middle-aged) |
| GSE35959-C-donor2（#353） | female | 67 yrs (middle-aged) |
| GSE35959-C-donor3（#295） | male | 61 yrs (middle-aged) |
| GSE35959-C-donor4（#296） | female | 62 yrs (middle-aged) |
| GSE35959-C-donor5(#247) | female | 56 yrs (middle-aged) |
| GSE35959--old_donor1(#520) | female | 79 yrs (elderly-aged) |
| GSE35959-old_donor2(#559) | male | 79 yrs (elderly-aged) |
| GSE35959-old_donor3(#606) | female | 80 yrs (elderly-aged) |
| GSE35959-old_donor4(#663) | female | 89 yrs (elderly-aged) |
| GSE35959-osteopo_donor1(#535) | female | 79 yrs (elderly) |
| GSE35959-osteopo_donor2(#547) | female | 94 yrs (elderly) |
| GSE35959-osteopo_donor3(#558) | female | 87 yrs (elderly) |
| GSE35959-osteopo_donor4(#572) | female | 82 yrs (elderly) |
| GSE35959-osteopo_donor5(#573) | female | 89 yrs (elderly) |
| GSE35959-senescent_donor1(#276) | female | 42 yrs |
| GSE35959-senescent_donor2(#278) | female | 59 yrs |
| GSE35959-senescent_donor3(#271) | female | 64 yrs |
| GSE35959-senescent_donor4(#277) | male | 54 yrs |
| GSE35959-senescent_donor5(#274) | male | 63 yrs |
