# Supplementary material for: Comprehensive analysis of ferroptosis-related genes reveals potential therapeutic targets in osteoporosis patients: a computational analysis and in vitro experiments
Source: Front Genet. 2025 Jan 10;15:1522809. doi: 10.3389/fgene.2024.1522809 (PMC11757248; doi:10.3389/fgene.2024.1522809)
Supplement: Supplementary file 5 [file Table4.docx]

The samples were as follows: 6 females in the elderly osteoporosis group, 4 females and 2 males in the elderly normal group, 2 females and 4 males in the middle-aged normal group.

| Elderly-osteoporosis1 | 81yrs, female |
| --- | --- |
| Elderly-osteoporosis2 | 68yrs, female |
| Elderly-osteoporosis3 | 72yrs, female |
| Elderly-osteoporosis4 | 82yrs, female |
| Elderly-osteoporosis5 | 76yrs, female |
| Elderly-osteoporosis6 | 74yrs, female |
| Elderly-norma1 | 64yrs, female |
| Elderly-norma2 | 75yrs, female |
| Elderly-norma3 | 72yrs, female |
| Elderly-norma4 | 62yrs, male |
| Elderly-norma5 | 61yrs, female |
| Elderly-norma6 | 65yrs, male |
| Middle-aged normal1 | 43yrs, male |
| Middle-aged normal2 | 46yrs, male |
| Middle-aged normal3 | 59yrs, female |
| Middle-aged normal4 | 58yrs, female |
| Middle-aged normal5 | 44yrs, male |
| Middle-aged normal6 | 48yrs, male |
